# Web Application to Enable Online Social Interactions in a Parkinson Disease Risk Cohort: Feasibility Study and Social Network Analysis

**DOI:** 10.2196/51977

**Published:** 2024-05-24

**Authors:** Xiancheng Li, Aneet Gill, Pietro Panzarasa, Jonathan Bestwick, Anette Schrag, Alastair Noyce, Anna De Simoni

**Affiliations:** 1 School of Business and Management Queen Mary University of London London United Kingdom; 2 Centre for Preventive Neurology Wolfson Institute of Population Health Queen Mary University of London London United Kingdom; 3 Queen Square Institute of Neurology University College London London United Kingdom; 4 Centre for Primary Care Wolfson Institute of Population Health Queen Mary University of London London United Kingdom

**Keywords:** pilot studies, network analysis, Parkinson disease, risk factors, risk, risk cohort, social interaction, development, neurodegenerative disease, neurodegenerative, United Kingdom, feasibility, design, pilot, engagement, users, online forum, online network, online, regression analysis

## Abstract

**Background:**

There is evidence that social interaction has an inverse association with the development of neurodegenerative diseases. PREDICT-Parkinson Disease (PREDICT-PD) is an online UK cohort study that stratifies participants for risk of future Parkinson disease (PD).

**Objective:**

This study aims to explore the methodological approach and feasibility of assessing the digital social characteristics of people at risk of developing PD and their social capital within the PREDICT-PD platform, making hypotheses about the relationship between web-based social engagement and potential predictive risk indicators of PD.

**Methods:**

A web-based application was built to enable social interaction through the PREDICT-PD portal. Feedback from existing members of the cohort was sought and informed the design of the pilot. Dedicated staff used weekly engagement activities, consisting of PD-related research, facts, and queries, to stimulate discussion. Data were collected by the hosting platform. We examined the pattern of connections generated over time through the cumulative number of posts and replies and ego networks using social network analysis. We used network metrics to describe the bonding, bridging, and linking of social capital among participants on the platform. Relevant demographic data and Parkinson risk scores (expressed as an odd 1:x) were analyzed using descriptive statistics. Regression analysis was conducted to estimate the relationship between risk scores (after log transformation) and network measures.

**Results:**

Overall, 219 participants took part in a 4-month pilot forum embedded in the study website. In it, 200 people (n=80, 40% male and n=113, 57% female) connected in a large group, where most pairs of users could reach one another either directly or indirectly through other users. A total of 59% (20/34) of discussions were spontaneously started by participants. Participation was asynchronous, with some individuals acting as “brokers” between groups of discussions. As more participants joined the forum and connected to one another through online posts, distinct groups of connected users started to emerge. This pilot showed that a forum application within the cohort web platform was feasible and acceptable and fostered digital social interaction. Matching participants’ web-based social engagement with previously collected data at individual level in the PREDICT-PD study was feasible, showing potential for future analyses correlating online network characteristics with the risk of PD over time, as well as testing digital social engagement as an intervention to modify the risk of developing neurodegenerative diseases.

**Conclusions:**

The results from the pilot suggest that an online forum can serve as an intervention to enhance social connectedness and investigate whether patterns of online engagement can impact the risk of developing PD through long-term follow-up. This highlights the potential of leveraging online platforms to study the role of social capital in moderating PD risk and underscores the feasibility of such approaches in future research or interventions.

## Introduction

Parkinson disease (PD) is the second most common neurodegenerative disease after Alzheimer disease [1]. It is associated with disability, loss of independence, caregiver burden, and premature death [2]. Life expectancy is reduced for all patients, especially in individuals with early-onset PD [2]. More than 75% have dementia after 8 years from diagnosis [3]. PD onset age is typically 60-65 years [4], with early onset being considered between 21 and 50 years [5].

Treatments and interventions for PD are more likely to be effective if they are applied before the current point of diagnosis, by which more than 50% of brain cells have already been lost [6]. While neuroprotective agents for neurodegenerative disease have shown promise in experimental studies [7,8], clinical research on the prevention of PD strategies in “at-risk” individuals is relatively understudied [9].

Studies have suggested that lifestyle measures such as reducing stress and increasing social interaction may be protective factors for neurodegenerative diseases [10,11]. Social capital refers to any type of benefit that individuals or groups can achieve through their underlying social relationships [12]. Changes in social interactions (or variations in social capital) can be associated with variations in risk factors for early death, poor mental health, and development of dementia [13,14]. There are 3 types of social capital, known as bonding, bridging, and linking [15]. Bonding Social Capital is characterized by intragroup connections among members of a network who are similar [16], often in terms of sociodemographic characteristics such as family, age, or interests. Bridging Social Capital derives from ties across diverse otherwise disconnected groups, including individuals from varied communities or organizations [17]. Linking Social Capital bridges the gap between different power tiers, typically linking community members with influential individuals or institutions with substantial resources [18]. Several studies have investigated the association between social capital and health-related outcomes, including health care accessibility and the provision of health education services [19,20].

PREDICT-Parkinson Disease (PREDICT-PD) is a web-based study of the UK general population to identify a group at higher risk of PD, using an algorithm that estimates the risk of developing PD from information that can be collected using online tools before the symptoms appear [21]. More than 7700 people across the United Kingdom were enrolled in PREDICT-PD when undertaking annual tests online, with in-person assessments, such as brain scans, happening in a smaller proportion. Traditional methods of evaluating social interactions have predominantly depended on self-administered surveys by participants [22]. By leveraging the PREDICT-PD platform, we can directly observe participants’ social engagement through their forum posts and responses, enabling the analysis of the social involvement of a broader participant group. While several studies have shed light on the impact of social capital and social networks on the well-being of older individuals with chronic noncommunicable diseases [23] and those at risk for dementia [24], there is a limited body of research focusing specifically on the association between social capital and the risk of PD. A recent study found that social isolation was associated with greater patient-reported PD severity and lower quality of life. Participants who reported having an extensive circle of friends had 21% fewer symptoms compared to those with limited or no social connections [25]. This finding underscores a potential link between social capital and PD severity.

In this pilot study, we aimed to assess the digital social characteristics of people at risk of developing PD and their social capital within the PREDICT-PD platform. We used various network metrics to describe the bonding, bridging, and linking of social capital among participants on the platform [20]. Our objective is to make and assess hypotheses about the relationship between online social engagement and potential predictive risk indicators of PD, specifically in the context of social capital [20]. The insights gained from this pilot study can inform future research or interventions. Specifically, beyond the main aim to test the feasibility and acceptability of this approach, we set to test the following research questions: (1) whether the breadth of an individual’s social circle, as represented by degree centrality, positively or negatively correlated with the risk of PD; (2) whether the proximity of an individual to others in the network, as represented by closeness centrality, positively or inversely correlated with the risk of PD; and (3) whether a high local clustering coefficient, indicative of social embeddedness in a closely knit community, associated with an increased or decreased risk of PD.

## Methods

### Overview

This study used a web-based application built to enable social interaction through the PREDICT-PD portal. The online forum was made public in April 2021 for 4 months. Feedback on the web-based application was collated from 5 PREDICT-PD cohort “champions,” who were representatives of the PREDICT-PD participant focus group. Champions are participants of the PREDICT-PD study who work in partnership with the PREDICT-PD team through regular meetings, to ensure that the views of patients, caregivers, and the public are considered when decisions are made. They have been taking part in the study for up to 13 years.

This feedback informed our plan for launching the platform and strategy for engagement, that is, appointing dedicated staff to ensure the platform was successful and stayed focused on issues relevant to PREDICT-PD participants, with weekly engagement activities, consisting of PD-related research, facts, and queries, to stimulate discussion.

Data were collected by the hosting platform. PREDICT-PD cohort participants were sent an email about the forum and imminent pilot study. The option “Forum” appeared on the left-hand menu bar, and anybody could access the terms and conditions of engagement and register a username. Registered users could choose to either write posts publicly or send private posts. In the latter case, posts were shared between 2 users only, whereas when posts were written publicly, a large number of users could become connected through threads of posts. Only posts that were shared publicly were collected and analyzed. For this study, user IDs were anonymized, and no demographic information was collected. The data sets included posts and their metadata (ie, the anonymized user ID numbers), user roles (eg, user, administrator, or moderator), date of posting, the hierarchical level of the post within the corresponding thread, and the dates in which the users joined and left the community. The community was moderated by members of the research team.

### Data Analysis

We looked at the number of users, the number of posts and connections per user, and posting frequency. A connection (ie, a tie, link, or edge) was established from 1 user to another when the former replied to a post submitted by the latter. We examined the pattern of connections generated over time through the cumulative number of posts and replies. We were interested not just in the number of posts and responses but in who responded to whom and when. To this end, we used social network analysis [26] to visualize and study the structure of the relationships among users.

Both visualization and analysis were conducted using Python (Python Software Foundation). The network analysis was carried out through additional custom computer code in Python. Descriptive analysis of the networks (ie, number of users, posts, and posting frequency) was carried out using the *Pandas* library, an open-source library providing data structures and analysis tools for the Python programming language, and *NetworkX*, another library facilitating analysis and calculations of network metrics. Taking each user as a node and replies as links, we constructed a user network. We focused on the largest connected component of this network and used several measures including, first, degree, defined as the number of links connected to the node. A high degree suggests that the user is directly connected to many other users, indicating a higher volume of social interactions in the online health community . In the context of social capital, it represents the quantity of direct interactions or the breadth of a user’s social interactions.

The second measure was betweenness centrality, which measures the number of times a node lies on the shortest path (ie, a path between 2 nodes that has the smallest number of links) between other nodes. It indicates how often an individual acts as a “bridge” or intermediary between others within the network, connecting different parts or groups, thus representing a form of Bridging Social Capital. An individual with high betweenness centrality is key in facilitating the flow of information and resources across diverse segments of the network, thus enhancing the network’s overall diversity and integration.

The third measure was closeness centrality, which measures the average shortest distance (ie, the number of links in the shortest path) from each node to all other nodes in the network (thus indicating how close a node is to all other nodes in the network). A higher closeness centrality means that a user can reach many other users in fewer steps. In terms of social capital, this reflects how efficiently an individual can access or disseminate information within the community. An individual with high closeness centrality might be better positioned to gather diverse insights or support from the community.

Fourth, we also constructed ego-centered networks for each user and measured the local clustering coefficient, which quantifies the degree to which the user’s neighbors tend to cluster together. The local clustering coefficient assesses how interconnected an individual’s neighbors are. A high value suggests that many of the user’s contacts also interact with each other, creating a tightly knit community. In the context of social capital, it represents the intensity of social interactions within a user’s immediate circle, which aligns with the concept of Bonding Social Capital. A high clustering coefficient indicates density among a group, which might have implications for emotional and informational support [20].

Fifth, effective size, is calculated by the number of neighbors that the user has, minus the average number of ties that each of the neighbors have to other neighbors. The effective size quantifies the degree to which the user’s ego-centered network is rich in structural holes and thus provides the node with bridging potential and brokerage opportunities. Effective size can also be used as a measure of Bridging Social Capital. A higher value indicates more structural holes in the user’s ego-centered network and a higher bridging ability.

By using regression analysis, we aim to investigate the association between each of the above forms of participants’ social capital and their PD risks.

Relevant demographic data and Parkinson risk scores (expressed as an odd 1:x) were analyzed using descriptive statistics. Regression analysis was conducted to estimate the relationships between PD risk scores (after log transformation) and network measures using *statsmodels* (a Python module that provides classes and functions for the estimation of statistical models).

### Ethical Considerations

This study received ethical approval from the Queen Square Ethics Committee (Research Ethics Committee reference: 10/H0716/85; Integrated Research Application System project ID: 20769). Informed consent for participation, collection, and analysis of engagement data was sought before users joined the online community through the online platform. Usernames were replaced with numerical codes and analyses were performed on anonymized and aggregated data. The PREDICT-PD website uses encrypted data capture, and the Amazon Web Services server is ISO270001 accredited. Profile information and research data are stored separately.

## Results

### Participants

A total of 219 participants from a cohort of 7700 took part in the pilot forum, including 218 users and 1 administrator who posted a blog weekly. For the 218 users, age could be matched for 187 (85.8%) users, gender for 193 (88.5%) users, and PD risk score for 187 (85.8%) users with data previously collected from the PREDICT-PD database. The age and gender of forum participants were consistent with PREDICT-PD cohort members [21], with a slight predominance of female members (Table 1).

**Table 1:** Characteristics of participants in the largest component (n=200)^a^.

Sample characteristics			Values
Number of posts per user, mean (range)			1.60 (1-6)
Total number of posts (admin’s posts excluded), n			348
**Age (years)**			
	Known age, n (%)	172 (86)	
	Unknown age (missing from matched PREDICT-PD^b^ data), n (%)	28 (14)	
	Mean (range)	67.55 (25.84-83.60)	
**Gender**			
	Male, n (%)	80 (40)	
	Female, n (%)	113 (56.5)	
	Unknown (missing from matched PREDICT-PD data), n (%)	7 (3.5)	
**Risk of Parkinson disease**			
	Known risk, n (%)	172 (86)	
	Unknown risk (missing from matched PREDICT-PD data), n (%)	28 (14)	
	Risk score (log-transformed), median (IQR)	4.21 (2.10-6.39)	
**Network characteristics, mean (range)**			
	Degree	1.93 (1-18)	
	Closeness centrality	0.331 (0.005-0.417)	
	Betweenness centrality	0.006 (0-0.125)	
**Ego network characteristics, mean (range)**			
	Local clustering coefficient	0.082 (0-1)	
	Effective size	1.79 (1-17)	

^a^Analysis performed on 91.7% (200/218) of users connected in the largest component.

^b^PREDICT-PD: PREDICT-Parkinson Disease.

### Online Social Interactions

**Figure 1:**
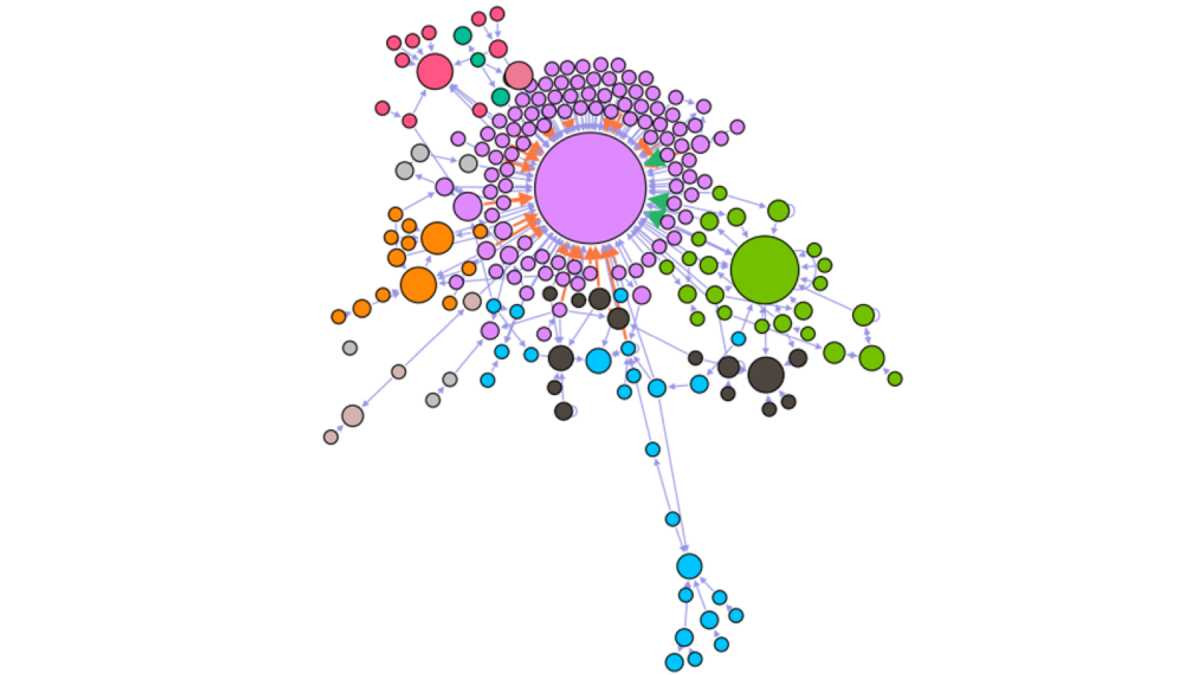
Cumulative network of individual participants in the largest component across the time span analysed. The size of the node correlates with the number of posts written by that user. Colors represent different groups of discussions.

Over 4 months, through posting to each other and the administrator, 91.7% (200/218) of users became part of a connected group (the largest component; see Figure 1). The largest community formed around the admin staff posting weekly (Figure 1; purple color), although smaller communities were started by users themselves (Figure 1; green, pink, orange, green, blue, and black color). Therefore, communities are formed independently from the actions of admin staff.

The characteristics of users in the largest component (200/218, 91.7%) have been summarized in Table 1.

**Figure 2:**
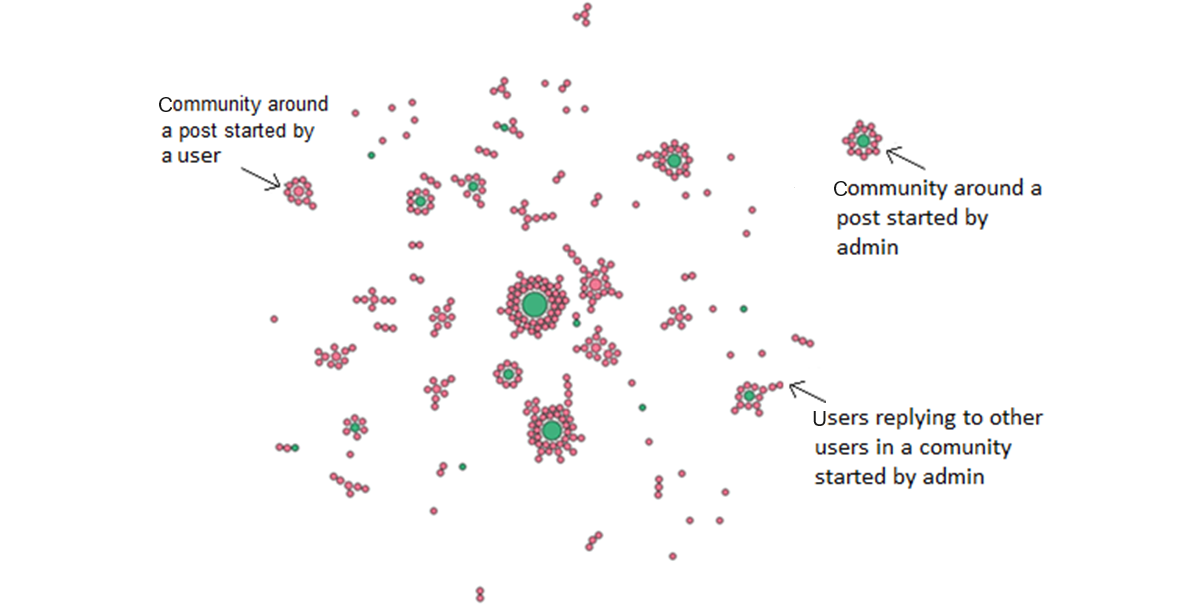
Representation of communities. Cumulative network of posts across the time span analysed. Circles represent individual posts: in green posts written by admin and in red posts by users.

Figure 2 illustrates the network of posts, where each node represents an individual post. Posts written by the admins are in green and posts by users are in red. The size of each circle is proportional to the number of replies received. Participants wrote a total of 364 posts, divided into 34 threads. Of the 34 threads, 14 (41%) were started by the administrator; 20 (59%) were spontaneously started by users, attracting a total of 133 posts, written by 99 individual users (see Figure 2). Since the start of the pilot, users created small communities of discussion, independently from the administrator. Weekly blogs were effective in creating conversations among users.

To show the development of discussion in the pilot forum, we measured the time gap between the original post and the replies in each thread. The frequency of replies with corresponding time gaps is plotted in Table 2. We found that participation was asynchronous (as is typical in online communities), with users replying to posts at different times. Most replies are written within a week after the original posts. The mean time gap between the reply and the original posts is 5.67 (SD 11.28) days. However, some users also replied to older posts with a maximum time gap of 82 days. This suggests that users were checking the forum and were motivated to participate in ongoing conversations. This pattern shows that the forum served not only as a platform for immediate exchange of information but also as a repository of knowledge and ideas that users revisited over time. These findings suggest a dual nature of the forum—as a space for timely interaction and as a valuable archive of discussions that continue to engage users over extended periods.

As network measures sometimes correlate with each other, we checked the correlations among the measures listed in Table 1. According to the correlation matrix in Table 3, betweenness centrality and effective size are significantly correlated with degree centrality (*P*<.001). To avoid multicollinearity, we did not include the 2 measures in regression models.

Regression results are given in Table 4. In model 1, we regressed the risk scores on the control variables’ age and gender. In models 2, 3, and 4, we added 3 network measures (ie, degree centrality, closeness centrality, and local clustering coefficient) separately in the regressions. In model 5, we added all 3 measures. The results showed a correlation that the association between Parkinson risk and degree centrality (ie, a high number of direct connections) is significant (see Table 4, models 2 and 5, *P*<.10). This may be related to the nature of the forum leading participants with lower PD risk scores (ie, higher risks of developing PD) to reach out for help and suggestions, which results in more discussion and communication with other participants.

The nonsignificant results on closeness centrality and local clustering coefficient offer preliminary insights into the direction of their associations with Parkinson risk. Local clustering coefficients tend to negatively correlate with the risk score, meaning that users with lower risk scores (ie, higher risks of developing PD) were more likely to embed themselves into tightly knit communities. This is possible because participants with higher PD risks may share similar experiences and show interest in similar topics within the forum. On the opposite, users with higher scores (ie, lower risks of developing PD) tended to display higher closeness centrality. This could suggest that users with closer access to many other members within the forum (ie, those who could reach out to a large portion of the forum and receive information very quickly without necessarily being the hubs) are less likely to have a higher risk of developing PD.

We performed tests for multicollinearity with variance inflation factor. Since each of the variance inflation factor values for the predictor variables in the model is close to 1, multicollinearity is not a problem in the model.

This finding suggests that users’ connections to others in the online community can be associated with the risk of developing PD, thus showing the potential of correlating network properties of users in online communities to the risk of diseases.

**Table 2:** Distribution of replies over time (days) after the first post in the corresponding thread.

Days	Posts, n (%)
0	131 (44.7)
1	37 (12.6)
2	14 (4.8)
3	27 (9.2)
4	7 (2.4)
5	7 (2.4)
6	4 (1.4)
7	8 (2.7)
8	2 (0.7)
9	6 (2.0)
10	1 (0.3)
11	3 (1.0)
12	2 (0.7)
14	4 (1.4)
15	1 (0.3)
16	3 (1.0)
17	1 (0.3)
19	2 (0.7)
20	1 (0.3)
21	3 (1.0)
23	2 (0.7)
24	6 (2.0)
25	5 (1.7)
26	1 (0.3)
31	1 (0.3)
33	8 (2.7)
34	1 (0.3)
44	2 (0.7)
67	1 (0.3)
73	1 (0.3)
82	1 (0.3)

**Table 3:** Correlation matrix.

Variable			Risk score	Age		Gender		Degree		Closeness		Betweenness		Clustering coefficient		Effective size
**Risk score**																
	*r*	1		–0.42	–0.32		–0.14		0.03		–0.21		–0.14		–0.13	
	*P* value	—^a^		<.001	<.001		.07		.69		.005		.07		.09	
**Age**																
	*r*	–0.42		1	0.15		0.05		0		0.05		0.11		0.03	
	*P* value	<.001		—	.06		.54		.99		.53		.17		.68	
**Gender**																
	*r*	–0.32		0.15	1		0.1		–0.09		0.14		–0.07		0.12	
	*P* value	<.001		.06	—		.17		.25		.06		.39		.12	
**Degree**																
	*r*	–0.14		0.05	0.1		1		0.11		0.84		0.13		0.98	
	*P* value	.07		.54	.17		—		.13		<.001		.09		<.001	
**Closeness**																
	*r*	0.03		0	–0.09		0.11		1		0.12		0.24		0.06	
	*P* value	.68		.99	.25		.13		—		.12		.002		.44	
**Betweenness**																
	*r*	–0.21		0.05	0.14		0.84		0.12		1		–0.03		0.85	
	*P* value	.005		.53	.06		<.001		.12		—		.74		<.001	
**Clustering coefficient**																
	*r*	–0.14		0.11	–0.07		0.13		0.24		–0.03		1		–0.04	
	*P* value	.07		.17	.39		.09		.002		.74		—		.64	
**Effective size**																
	*r*	–0.13		0.03	0.12		0.98		0.06		0.85		–0.04		1	
	*P* value	.09		.68	.12		<.001		.44		<.001		.64		—	

^a^Not applicable.

**Table 4:** Logistic regression models showing the predictors of Parkinson disease risk^a^.

Parameter	Model 1			Model 2			Model 3			Model 4			Model 5	
	B (robust SE)	*P* value	B (robust SE)		*P* value	B (robust SE)		*P* value	B (robust SE)		*P* value	B (robust SE)		*P* value
Intercept	15.64^b^ (1.56)	<.001	15.84^b^ (1.54)		<.001	15.52^b^ (1.80)		<.001	15.40^b^ (1.58)		<.001	14.90^b^ (1.75)		<.001
Age	–0.16^b^ (0.02)	<.001	–0.16^b^ (0.02)		<.001	–0.16^b^ (0.02)		<.001	–0.15^b^ (0.02)		<.001	–0.15^b^ (0.02)		<.001
Gender	–1.65^b^ (0.44)	<.001	–1.59^b^ (0.44)		<.001	–1.64^b^ (0.44)		<.001	–1.71^b^ (0.43)		<.001	–1.63^b^ (0.44)		<.001
Degree	—^c^	—	–0.16^d^ (0.08)		.06	—		—	—		—	–0.14^d^ (0.09)		.097
Closeness	—	—	—		—	0.36 (2.98)		.90	—		—	2.04 (2.96)		.49
Clustering coefficient	—	—	—		—	—		—	–1.47 (1.03)		.15	–1.46 (1.05)		.16
Observation, n	172	—	172		—	172		—	172		—	172		—

^a^This analysis includes the 86% (172/200) of forum users in the largest component, whose data about age, gender, degree, closeness, and clustering coefficient could be matched from the PREDICT-Parkinson Disease database.

^b^*P*<.01.

^c^Not applicable.

^d^*P*<.10.

## Discussion

This 4-month pilot showed that a forum application within the PREDICT-PD web platform was feasible and acceptable, and fostered digital social interaction. Participants’ activity over time showed that the forum was used as a repository of knowledge and ideas that users revisited over time.

Forum participants’ characteristics were consistent with PREDICT-PD cohort members. Matching with previously collected PREDICT-PD data was feasible, showing a potential for future analyses correlating online network characteristics with the risk of PD over time, as well as testing digital social engagement as an intervention to modify the risk of developing neurodegenerative diseases.

Engagement with the forum was found to take place independently from the weekly engagement activities of admin staff. As more participants joined the forum and connected to one another through online posts, distinct groups of connected users started to emerge. These groups have fundamental implications for the effectiveness of processes of network dynamics such as information diffusion [27]. In the 4-month pilot, the community underwent the formation of the “largest component” of connected users. It is well known that components are critical for information diffusion [28,29]. Without a large, connected component, users would be members of small, isolated islands, and information would be unable to flow from 1 island to another.

Based on the regression results, we hypothesize that users with lower risk scores (ie, higher risk of developing PD) may be those reaching out for help and suggestions within the forum, with a tendency to display a higher degree of centrality. Conversely, users who keep being active in the central community (ie, with higher closeness centrality) tend to have higher risk scores (ie, lower risk of developing PD). In this exploratory analysis of the associations between network characteristics and PD risk, some of the findings did not reach statistical significance. This outcome, while representing a limitation, is not unexpected given the pilot nature of the study, designed primarily for feasibility and methodological exploration rather than definitive causal inference. These preliminary findings serve as an important stepping stone, highlighting the need for larger, more comprehensive studies. Future research should build on this foundational work, using larger sample sizes and longer durations to adequately power the investigation of the potential impact of network characteristics on PD risk.

These results suggest that the online forum could serve as a platform for targeted interventions for individuals with higher PD risks to spontaneously gather and engage with one another. By monitoring network measures linked to their engagement, we might be able to identify users with higher PD risk and provide them with tailored resources or direct interventions.

This pilot study adopted an observational research approach. An important limitation inherent to such studies is the possibility of reverse causation influencing the detected associations, making it essential to consider the duration of follow-up. Specifically, we observed that local clustering coefficients tend to negatively correlate with the risk score, suggesting that individuals at higher risk of developing PD might preferentially engage in tightly knit communities. This could reflect a tendency among participants with higher PD risks to seek out and share experiences with peers facing similar challenges. Conversely, it is also possible that the formation of such tightly knit communities and the homogeneous nature of the information exchanged within them could, if predominantly negative, contribute to increased stress or negative emotional states, potentially elevating the risk of PD. This dual interpretation underscores the complexity of the reverse causation issue, as it illustrates how the detected associations could be interpreted differently. A deeper understanding can be gained by analyzing patterns of engagement in social activities over time and correlating them with the risk of PD. This can be facilitated using data from studies with repeated assessments of social participation and associated risk metrics, which will be the next step of this research.

Future research based on a larger data set would allow for more robust statistical analysis, potentially confirming the trends observed in this pilot study, and would allow us to investigate whether active online participation can be associated with modification of PD risk scores and improved health outcomes, or whether it rather reflects a self-selection bias prompting individuals with higher risks to engage in forum discussions more intensely. Longitudinal studies could also determine whether changes in network centrality measures correlate with changes in users’ health status in the context of neurodegenerative diseases. Such studies will aim to use advanced statistical models such as mixed effects models or growth curve analysis to discern patterns of change and causality. Specifically, it would be insightful to investigate how early changes in social network dynamics precede the onset of PD symptoms, and whether increasing social connectivity can mitigate risk. Besides, by using longitudinal data, temporal centrality measures can be calculated, and dynamic network analysis can significantly enhance the depth and breadth of insights, especially in tracking changes in social engagement and their impact on PD risk.

Additionally, qualitative analysis of the content of communications could provide deeper insights into the types of support and information being exchanged [20] and how these might relate to PD risk and management. Moreover, qualitative methodologies could enable us to uncover factors contributing to users’ engagement with the forum, particularly when taking place independently of the admin staff’s activities, for example, specific user-driven initiatives or topics that lead to increased participation. Understanding these dynamics could provide insights for future digital social interventions targeting people at risk of developing PD.

The results presented here show the potential of an online forum as an intervention to trigger social connectedness and to investigate whether patterns of online engagement [20] have a causal impact on the risk of developing PD through long-term follow-up.

## Abbreviations

PD: Parkinson disease
PREDICT-PD: PREDICT-Parkinson Disease

## Data Availability

The data that support the findings of this study are available from the corresponding author upon reasonable request.
